# Colonic Metastasis of Renal Cell Carcinoma: A Rare Cause of Lower Gastrointestinal Bleeding

**DOI:** 10.7759/cureus.91037

**Published:** 2025-08-26

**Authors:** Daniel J Canter, Urmimala Chaudhuri, Chase Thornton, Hassan Zreik, Drew Triplett

**Affiliations:** 1 Internal Medicine, Wright State University Boonshoft School of Medicine, Dayton, USA; 2 Gastroenterology, Wright State University Boonshoft School of Medicine, Dayton, USA

**Keywords:** gi metastasis, lower gi bleed, lower gi endoscopy, metastasis to the colon, procedures endoscopic, renal cell metastasis

## Abstract

Renal cell carcinoma (RCC) metastasis to the large bowel is exceptionally rare and can present as lower gastrointestinal bleeding. We report the case of an 80-year-old man who presented to our institution with a history of left-sided RCC and known pulmonary and osseous metastases, who presented with recurrent rectal bleeding and profound anemia. Initial colonoscopy demonstrated an ulcerated splenic-flexure nodule, but biopsies were non-diagnostic. One week later, flexible sigmoidoscopy revealed a friable, actively bleeding rectal lesion; endoscopic polypectomy and hemostatic clips were placed. Histopathology confirmed metastatic RCC. Given his advanced disease and functional status, goals-of-care discussions resulted in the initiation of comfort care. This case underscores the importance of maintaining a broad differential for lower GI bleeding in patients with known RCC, including colonic metastasis. Endoscopic evaluation not only facilitates diagnosis but also provides therapeutic hemostasis. A multidisciplinary approach combining gastroenterology and oncology may help ensure accurate diagnosis, effective management, and alignment of care with patient-centered goals of care.

## Introduction

Renal cell carcinoma (RCC) is the ninth most common malignancy in the United States and had a global incidence of over 434,000, according to Globocan 2022 [1]. RCC accounts for the vast majority of primary renal malignancies and is further classified based upon its histopathological makeup, the most common subtypes being clear cell RCC and papillary RCC [2].

Clinically, RCC may present with hematuria, flank pain, or a palpable abdominal mass but may also be discovered incidentally on imaging [3]. Around one-fifth to one-third of RCC cases are estimated to be metastatic at the time of diagnosis [4]. Metastasis of RCC is most commonly hematogenous, most often to the lungs, lymph nodes, bone, and liver. RCC has more rarely been reported to metastasize to the colon and rectum of the gastrointestinal (GI) tract, typically in the context of widespread metastatic disease involving other organ systems [5]. Solitary metastasis of RCC to the colon is exceedingly rare, with only 12 documented cases worldwide as of a 2019 case report and accompanying literature review [6]. RCC metastasis to the rectum or anal canal is even less common, with just eight cases identified in a separate 2019 literature review [7]. Typical treatment of metastatic RCC is usually accomplished with systemic therapy, including tyrosine kinase inhibitors or targeted immunotherapy, with specific therapy dependent upon tumor biology and patient factors [8]. There is limited literature regarding the treatment of RCC metastatic to the GI tract due to its rare nature. Here, we present a rare case of metastatic RCC with involvement of both the colon and rectum, highlighting diagnostic and management challenges that necessitate multidisciplinary management in uncommon presentations of patients with metastatic disease.

## Case presentation

**Figure 1:**
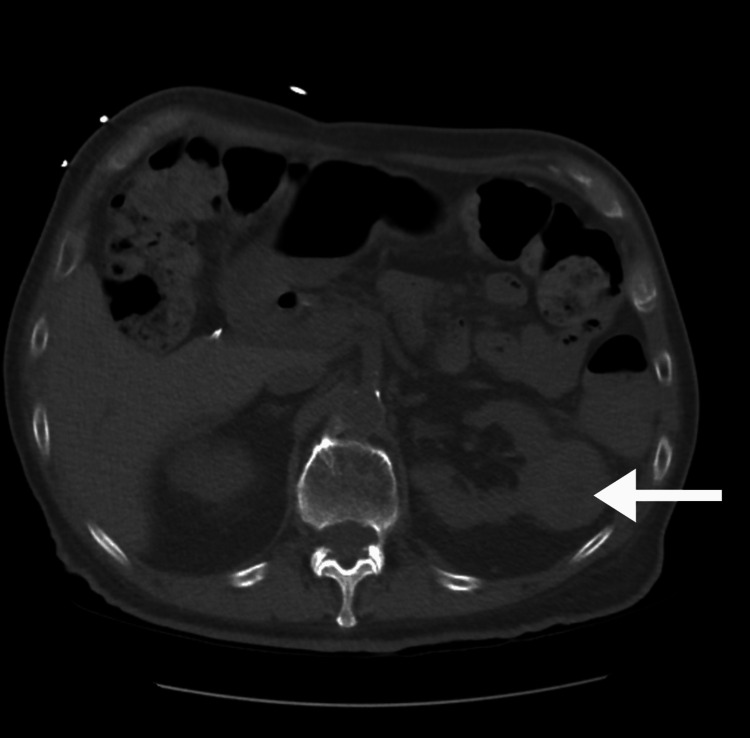
Computed tomography of the abdomen and pelvis showing a solid left renal mass (white arrow).

**Figure 2:**
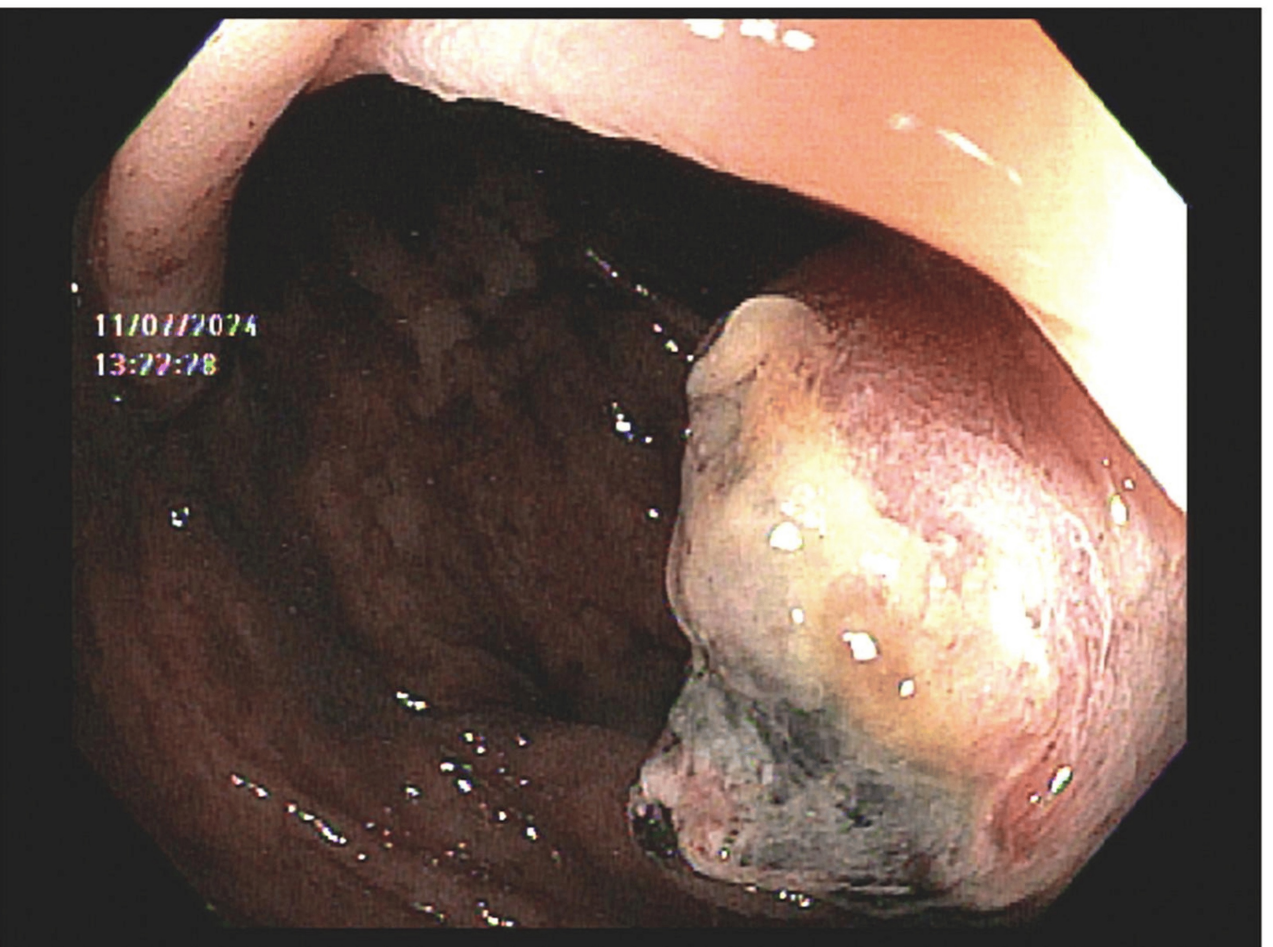
Ulcerated splenic-flexure nodule on initial colonoscopy.

An 80-year-old man with a history of left-sided clear cell RCC metastatic to the lung and bone presented to our institution with a three-week history of intermittent rectal bleeding, 40 pounds of unintentional weight loss over the preceding three months, and constipation. On admission, his hemoglobin level was 6.0 g/dL. Additional laboratory values are displayed in Tables 1-2. Vitals at presentation were significant for hypotension (81/48 mmHg). Abdominal examination was benign: soft, non-distended, non-tender, with normal bowel sounds and no peripheral edema. Computed tomography of the abdomen and pelvis without contrast confirmed the known left renal mass and revealed new wall thickening at the splenic flexure, as shown in Figure 1. Gastroenterology was consulted, and a colonoscopy was performed, which demonstrated a dilated, tortuous colon and a 2-3 cm ulcerated nodule near the splenic flexure, as shown in Figure 2. Biopsies showed ulceration without malignancy at that time. Retroflexion in the rectum was not performed due to poor visualization.

**Table 1:** Key metabolic panel values at initial admission, discharge, readmission, and final assessment. BUN, blood urea nitrogen; eGFR, estimated glomerular filtration rate

Test	Reference range	Initial admission	Discharge	Readmission	Final assessment
Sodium (mEq/L)	135-148	133	138	133	139
Potassium (mEq/L)	3.4-5.3	5.1	4.5	4.9	4.8
Chloride (mEq/L)	96-110	98	106	97	105
Carbon dioxide (mEq/L)	19-32	24	24	23	24
Glucose (mg/dL)	70-99	491	102	458	130
BUN (mg/dL)	3-29	41	26	49	34
Creatinine (mg/dL)	0.5-1.4	2.4	2	2.2	1.9
BUN/creatinine ratio	7-25	17	13	22	18
Calcium (mg/dL)	8.5-10.5	8.7	7.8	8.9	8.2
Albumin (g/dL)	3.5-5.2	3.2		2.8	2.5
Phosphorus (mg/dL)	2.1-4.3				3.3
eGFR (mL/min/1.73 m²)	>=60	27	33	30	35
Anion gap	5-15	11	8	13	10

**Table 2:** Key CBC values at initial admission, discharge, readmission, and final assessment. CBC, complete blood count; WBC, white blood cell; RBC, red blood cell

Test	Reference range	Initial admission	Discharge	Readmission	Final assessment
WBC (K/uL)	3.5-10.9	11.6	9.8	12.8	11.6
RBC (M/uL)	4.14-5.80	2.03	2.65	1.99	2.95
Hemoglobin (g/dL)	13.0-17.7	7.2	7.6	6.4	8.6
Hematocrit (%)	37.5-51.0	22.1	23.9	20.3	26.3
Platelet count (K/uL)	140-400	320	297	341	282

**Figure 3:**
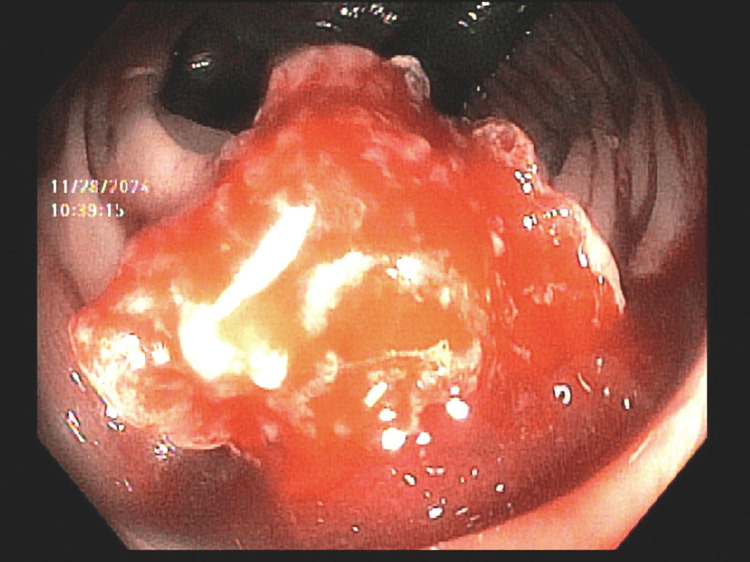
Actively bleeding, friable, ulcerated lesion in the rectum during flexible sigmoidoscopy.

He returned one week later with recurrent bleeding and a hemoglobin of 5.5 g/dL. On re-evaluation, he was hemodynamically stable (115/56 mmHg) and in no acute distress. Abdominal exam remained unremarkable, but the patient had overt signs of bleeding, including hematochezia and a guaiac-positive rectal exam. At this time, a flexible sigmoidoscopy identified a friable, ulcerated rectal nodule with multiple active bleeding points, as seen in Figure 3. Hemostasis was achieved with epinephrine injection, hot-snare polypectomy, and two hemoclips. Histopathology and immunostains of the resected tissue confirmed metastatic RCC with high-grade features, but were not available for inclusion in this case presentation. After multidisciplinary goals-of-care discussions, he elected for comfort care and expired shortly thereafter.

## Discussion

Metastasis to the large bowel in RCC is uncommon, with only 14 previously documented case reports of rectal metastases. Treatment approaches for RCC have evolved significantly in recent decades. Historically, radical nephrectomy was standard for localized disease, but partial nephrectomy, active surveillance, and ablation are now viable alternatives for selected patients based on disease severity, staging, and individual comorbidities [9,10]. The role of neoadjuvant and adjuvant therapies, such as immune checkpoint inhibitors, has yielded mixed outcomes in localized RCC [11]. For metastatic RCC, combination systemic therapy involving immune checkpoint inhibitors and tyrosine kinase inhibitors is the mainstay of treatment and has led to improved patient outcomes. Additionally, stereotactic body radiation therapy also plays a role in treatment [12].

For non-metastatic or localized RCC, surgical resection remains the standard; however, approximately 20%-50% of patients develop metastatic disease despite successful resection [1]. Therefore, careful surveillance across all stages of RCC is important. The National Comprehensive Cancer Network guidelines recommend tailored surveillance regimens based on factors such as disease stage, initial treatment modalities, and patient-specific variables [13]. For patients with stage IV disease or recurrent, unresectable disease, periodic imaging and laboratory monitoring are crucial. Surveillance imaging typically includes CT or MRI of the chest, abdomen, and pelvis, initially performed to establish baseline disease burden before therapy initiation. Follow-up intervals generally range from two to three months initially, with progression to every three to six months if disease remains stable, but this can vary based on disease aggressiveness, metastatic burden, and clinical judgment [8]. Baseline and annual head imaging with MRI is also suggested, with intervals adjusted according to clinical progression. MRI of the spine and bone scans may be considered if clinically indicated [9,13].

In the presented case, our patient had an established diagnosis of metastatic RCC, previously identified at typical metastatic sites. The patient had not pursued aggressive systemic therapy before presentation. With the development of new-onset rectal bleeding in the context of known metastatic RCC, GI metastasis was strongly suspected and ultimately confirmed by biopsy. Metastatic RCC involving the rectum is notably rare; however, symptomatic bleeding remains a common presenting symptom in nearly all documented cases [7]. Endoscopically, RCC metastases to the GI tract in our patient demonstrated highly friable, ulcerated nodules. His findings support the importance of careful endoscopic management in patients with potential GI metastasis.

The prognosis for RCC varies significantly by stage at initial diagnosis, with stage IV disease having a markedly poorer 5-year survival rate of approximately 12%, compared to approximately 93% for stage I disease [14]. Given our patient's advanced metastatic stage at the time of GI involvement, his prognosis was notably poor. Further curative treatments were not feasible, and discussions focused primarily on symptomatic and supportive measures. Consistent with the patient's wishes and clinical status, a comfort-focused approach was selected.

This case emphasizes the clinical importance of recognizing rare metastatic presentations of RCC, particularly those involving the GI tract in patients presenting with lower GI bleeding. Consistent with previously documented reports, symptomatic bleeding remains a hallmark of RCC metastasis to the large bowel or rectum [14-16]. This case reinforces the generally poor prognosis associated with advanced metastatic RCC, underscoring the critical role of patient-centered discussions regarding goals of care.

## Conclusions

Colonic metastases of RCC are an uncommon etiology of lower GI bleeding, and only 14 documented cases have reported rectal involvement before this case report. Early endoscopic evaluation facilitates timely diagnosis and hemostasis. Accurate diagnosis in this case was complicated by the need for repeated endoscopic assessments. Such presentations underscore the necessity of a multidisciplinary approach and clinical equipoise in effectively managing these uncommon manifestations.
